# Gambling Harm as a Global Public Health Concern: A Mixed Method Investigation of Trends in Wales

**DOI:** 10.3389/fpubh.2020.00320

**Published:** 2020-07-22

**Authors:** Bev John, Katy Holloway, Nyle Davies, Tom May, Marian Buhociu, Alecia L. Cousins, Samantha Thomas, Gareth Roderique-Davies

**Affiliations:** ^1^Addictions Research Group, Faculty of Life Sciences and Education, School of Psychology and Therapeutic Studies, University of South Wales, Pontypridd, United Kingdom; ^2^Substance Use Research Group, Faculty of Life Science and Education, Centre for Criminology, University of South Wales, Pontypridd, United Kingdom; ^3^Faculty of Health, School of Health and Social Development, Deakin University, Melbourne, VIC, Australia

**Keywords:** gambling harm, public health, mixed methods, gambling density, prevention, predictors of harm

## Abstract

**Background:** Recent research evidence has suggested that gambling is a public health concern. A number of studies report the association between gambling activity and increased instances of various other harms, including substance misuse and psychological disorders. In parallel to alcohol misuse, it is also becoming clear that gambling related harm is more of a continuum of harm, as opposed to traditionally accepted categorisations of gambling behavior: safe and responsible or “problem” and harmful. Previous effective treatment models for alcohol misuse have considered a public health approach to develop interventions. As such, the current research seeks to use a public health approach to both investigate the extent of gambling harm across Wales, and to identify upstream predictors of harm to inform future interventions.

**Method:** A triangulation of data collection methods was utilized across Wales, UK. Two hundred and forty-eight participants completed a quantitative survey relating to gambling behavior and related harm, which included the Problem Severity Gambling Index, the Gambling Commission measure of frequency, The Gambling Motives Questionnaire and the Fast Alcohol Screening tool. Ninety-eight of these participants completed a qualitative subsection. Structured interviews were conducted with 20 individuals from 11 service providers. Semi-structured interviews were conducted for the five case studies of individuals who had previously sought help for gambling. The geographical density and distribution of Licensed Gambling Outlets was also mapped in local areas.

**Results:** The findings provide further evidence of a continuum of gambling related harm. Twenty seven percent of survey participants demonstrate some indicators of risk of gambling harm. Social, cultural and environmental contexts play a role in initiation and maintenance of gambling behavior and the subsequent related harm. Accounts from individuals corroborated the quantitative findings.

**Conclusions:** Findings from this Welsh sample are in line with and add support to the growing international research evidence that gambling harms are a universal issue that cross cultures. It is clear that action is needed by legislators at a policy level and that broadening the focus of intervention to a public health level is necessary to develop effective strategies for harm reduction.

## Introduction

Gambling is increasingly being described as an important public health issue (1, 2). Gambling activity is often linked to other harms such as higher instances of substance misuse (3), greater likelihood of perpetrating intimate partner violence (4), greater risk of homelessness (5), and psychological disorders, specifically anxiety and depression (6). The UK Gambling Commission's recent telephone survey suggests that 4.2% of respondents may be at some risk of harm. However, as this survey uses a short screen version of the PGSI, they recommend referring to the full 2016 Health Survey findings, which identified the risk level as 0.7% of the population (7). As such, much focus has been on pathologies within individual gamblers, including problems such as impulsivity, lack of self-regulation and inability to “gamble responsibly.” However, evidence is emerging which demonstrates that risk of harm could be much higher; a recent Australian study (8) found that ~40% of participants were at some risk of gambling-related harm, although the authors note that the use of an online panel produced an over-representation of gamblers in this study.

Focusing on individual problems suggests a false dichotomy of “safe, social, or responsible” gambling on one side and “problem, dependent or pathological” gambling on the other. It is apposite here to consider the development of effective models of public health approaches to alcohol related harm, as, with both of these risk behaviors, it is increasingly evident that harm occurs on a continuum. In a seminal publication on approaches to alcohol treatment, Heather and Stockwell (9) highlight the significance of the emergence of harm prevention as an intervention strategy for alcohol misuse, which acknowledges the status of alcohol as a legal pastime engaged in by many people, a position also applicable to gambling. A parallel and related development was what Heather (ibid. p 5) coined a “broadening of the base of treatment,” which has subsequently underpinned a public health approach of prevention and early intervention to alcohol related harm. The relevance to gambling here is the acknowledgment of a continuum of harm and associated possibilities of “upstream” prevention, in place of the current focus on a minority of “dependent gamblers.” In an effort to broaden the definition and focus of gambling harms, Langham et al. (10) developed a comprehensive taxonomy, the breadth of which demonstrates a continuum across biopsychosocial contexts. This is also reflected in the research literature exploring the social, cultural and environmental contexts of gambling harm, an extensive review of which is beyond the scope of the current paper.

Babor et al. (11) suggest that whilst public health approaches usually focus on total populations, these may be more appropriately targeted at sub populations due to heterogeneity within gender, age and professional groups. This is illustrated by a recent study of the Welsh population that mapped potential gambling hotspots (12), which found two different geographical districts of the same city with demographically very distinct but equally risky gamblers. One was a run-down inner-city suburb with a high density of traditional Licensed Gambling Outlets (LGOs); the other was an area close to the university with a high concentration of students whose gambling is more likely to involve new online technologies. Such emerging gambling products and the changing means of access to gambling are intensifying differences in risky gambler profiles (13). These factors should be considered as potentially important contextual drivers of harm. This is further reflected in the rapidly changing environment in terms of advertising exposure and its impact that is being highlighted within the international literature, specifically on young people (14) and gambling populations more generally [for example, (15, 16)].

The importance of addressing gambling harm has been highlighted by a number of international studies [for example, (17–19)]. Although many of the identified issues may be universal, it is important to investigate and compare across geographical areas/cultures in order to inform policy makers with evidence within their own legislative regions. Gambling is a legal activity in many countries, usually regulated by non-health governmental departments, such as the Department for Digital, Culture, Media and Sport in the United Kingdom. As a country within the UK, the Wales Government has a number of devolved powers, including health, and the Chief Medical Officer (CMO) for Wales recently called for the Welsh Government to make a shift from an individual to a population led focus to tackle gambling harms (20).

The implementation of an effective public health approach necessitates the collection and synthesis of evidence that contextualizes the lived experiences of the individual gambler, within their wider social and cultural community, and alongside the commercial drivers and political contexts. A majority of the research focuses on the pathological gambler at the end of the continuum of harm, leaving an absence of data in the UK relating to the harms and predictors of harm across the gambling continuum, including horizon scanning for future problems. Evidence is needed for the development of effective harm reduction strategies at all points, including prevention, screening and evidence-based interventions.

The development of a public health framework has a number of specific steps, as defined by the World Health Organization [WHO; (21)] including: (1) understanding the nature and extent of the problem, (2) identifying causes, (3) developing and testing evidence-based means of resolving the problem, and (4) implementing solutions at a wider scale. The overall objective of the current study is to investigate steps 1 and 2 in relation to gambling harms, in order to provide evidence for steps 3 and 4, and thus contribute to the international literature on gambling harms and the possibilities for a public health approach to their amelioration.

Based on the research literature and the WHO public health framework as set out above, a multi-method approach (including survey, structured interview, and case study approaches) was utilized. The aims were: to investigate individuals presenting in treatment programmes and other services; to explore the broader, “upstream” gambling patterns and trends; and to map the geographical density of and access to gambling outlets. The study was guided by the following research questions:

1. What are the current patterns, trends and risks in gambling behavior?
2. What are the salient social, cultural and environmental factors in the development of gambling problems?
3. Do the personal accounts of individuals (gamblers and their families/friends at the problem-gambling end of the scale) have salience with patterns and trends in terms of risk predictors?
4. How effective are support services in identifying and helping individuals experiencing gambling harm?

## Methods

A triangulation of data collection approaches was utilized to achieve the project objectives (as set out above). These were:

### Online Survey

The survey consisted of four measures and a qualitative subsection:

- The Problem Gambling Severity Index (22) is a nine-item measure of problem gambling.
- Frequency and type of gambling behavior was measured using an adapted version of the Gambling Commission measure (2016).
- The Gambling Motives Questionnaire (23) is a 15-item measure of three motives for gambling; for excitement; for social reasons; as a coping strategy.
- The Fast Alcohol Screening tool (24) is a brief measure of hazardous drinking (often a comorbid risk factor with gambling).
- A qualitative section at the end of the survey allowed respondents to expand on their own experiences of gambling related harm, and the wider impact of gambling on themselves, their family and friends or the broader community. This was facilitated by a free text box with no word limit.

#### Sample and Recruitment of Survey Participants

The target population consisted of residents in Wales living within one of the five designated locations (Rhondda Cynon Taf, Vale of Glamorgan, Llandudno, Wrexham and Newport) who were over 18 years old (the legal age for gambling in Wales), had any experience of gambling, and were able to complete self-report questionnaires. These locations broadly reflected the constituency areas of the Welsh Government Assembly Members who co-funded this research. A weblink to the survey was distributed using Facebook and Twitter. The nature of the distribution method led to responses from outside of the targeted areas being collected. All responses from individuals indicating a Welsh residential postcode were included in the study.

Two hundred and forty-eight participants completed the survey. Approximately 60% female; 40% male; mean age 45.76 (SD 14.62), range 18–77, median 47. Ninety-six of these participants contributed to this qualitative section of the survey.

### Interviews With Providers of Services

Structured interview schedules were developed in order to ensure consistency of questions. Questions focused around knowledge and experiences of gambling harms; approaches to identifying and assisting individuals; and signposting and referring. A range of service providers were recruited to participate in the interviews. All participants worked for organizations that deal with homelessness, drug and alcohol use, domestic abuse, and financial debt issues. All services were Third/Voluntary Sector organizations and were geographically representative of the population under investigation. Twenty individuals from 11 organizations were recruited with respondents including a CEO, an Operations officer, Counselors, Debt Advisors and Caseworkers.

### Individual Case Studies

In-depth interviews were conducted with individuals who had sought help for their gambling from specific services (listed below). A semi-structured interview schedule was utilized to maintain consistency in the areas explored and to allow individual narratives to be recounted. The broad focus of the interview schedule related to questions to facilitate individual accounts of: initiation experiences and trajectories of gambling behavior; impact(s) on individual health and wellbeing (within a broad biopsychosocial context); help-seeking behavior and motivations to change.

Five individuals volunteered to participate:

- ***Participant one*** *had received support from a peer mentoring drop-in service for veterans*.
- ***Participant two*** *had received support from an organization that provides support for problem gamblers over the phone, on-line and in face-to-face counseling sessions*.
- ***Participant three*** *had received support from a regional Citizens Advice with a specific gambling advice service*.
- ***Participant four*** *had received support from a regional Citizens Advice with a specific gambling advice service*.
- ***Participant five*** *had received support from Gamblers Anonymous and then a specialist recovery service in South Wales*.

### Mapping Exercise of Gambling Outlet Density

A quantitative investigation of local density and availability of gambling outlets of 5 local authorities were mapped onto socio-economic and demographic indices. This consisted of two stages. The first was to obtain data regarding the availability and density of LGOs permitted to hold Fixed Odds Betting Terminals (FOBTs)^1^, within the five fieldwork sites representative of the population under investigation (Denbighshire, Newport, Rhondda Cynon Taff, Vale of Glamorgan and Wrexham). LGOs permitted to hold FOBTs include Bookmakers, Adult Gaming Centers (AGCs) and Bingo halls.

Freedom of Information requests were sent to the relevant licensing authorities^2^ requesting the postcodes of LGOs in each fieldwork location. The density of LGOs within each town center was then able to be mapped by locating the number of LGOs within a 400 m radius. Although there is no standard definition for what constitutes “high density,” we have followed previous research that indicates a 400 m (0.25 miles) radius around a LGO represents an accessible distance for anyone to move between outlets located within this radius (25).

The second stage was to highlight the socio-economic characteristics of the areas in which the LGOs were located. This was established by matching the postcode of the LGO location to its respective Lower Super Output Area (LSOA). LSOAs are small geographic areas that are consistent in population size (unlike wards) and are therefore easier to compare. In Wales, 1909 LSOAs exist. Each LSOA is scored on seven measures of deprivation (Income, Employment, Housing, Health, Education, Crime and Living Environment; Ministry of Housing and Local Government, 2019) and ranked from 1–1,909 (one being the most deprived) on the Welsh Index of Multiple Deprivation score (26). The WIMD is the official measure of relative deprivation in Wales.

### Data Analysis: Survey Data

Data were analyzed using the Statistical Package for Social Sciences (SPSS 2017) software (IBM Corp) (27). Descriptive and frequency statistics were used to describe the sample characteristics and gambling patterns. Analysis of Variance (ANOVA) was used to examine differences between gender, age and drinking status and gambling risk. Pearson correlation coefficients were calculated to establish relationships between all key variables. Multiple Regressions were conducted to test specific drivers or predictors of harm.

### Qualitative Data

The qualitative data of the survey and of the case study relates to individual accounts of lived experiences; thus, a thematic analysis of the data was conducted, with the objective of establishing patterns within the narratives. A content analysis was conducted as the most appropriate method for synthesizing this structured interview data from the service providers. Codes were then grouped together within overarching themes as presented below.

#### Qualitative Survey Data

**Figure 1:**
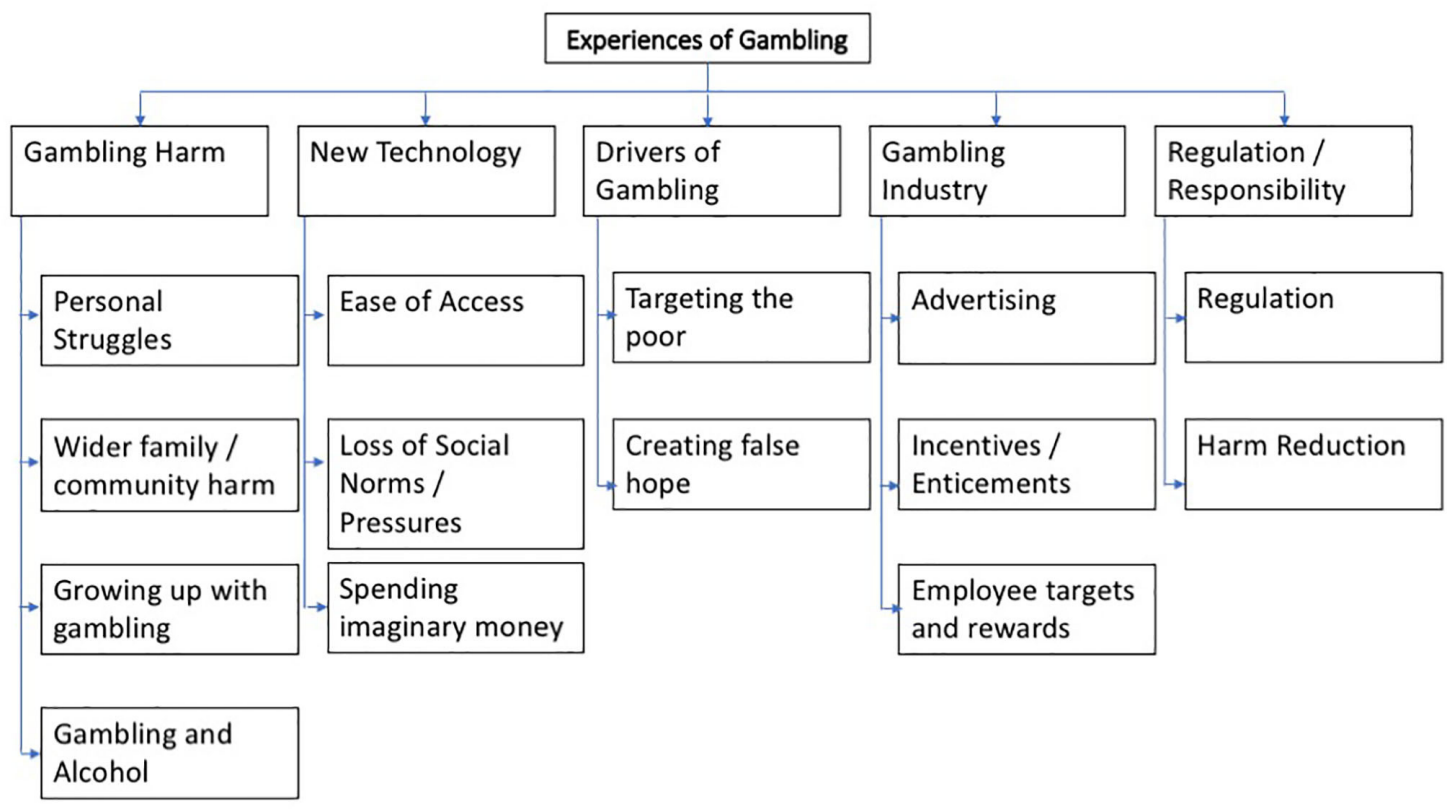
Overarching and sub-themes from qualitative survey data.

Five overarching themes were identified from the qualitative survey data, with subthemes clustered under each as set out in Figure 1 below. Findings which are relevant to the research questions are addressed within the results which are presented as “verbatim quotes” using the code “QS.”

#### Service Provider Interview Data

**Figure 2:**
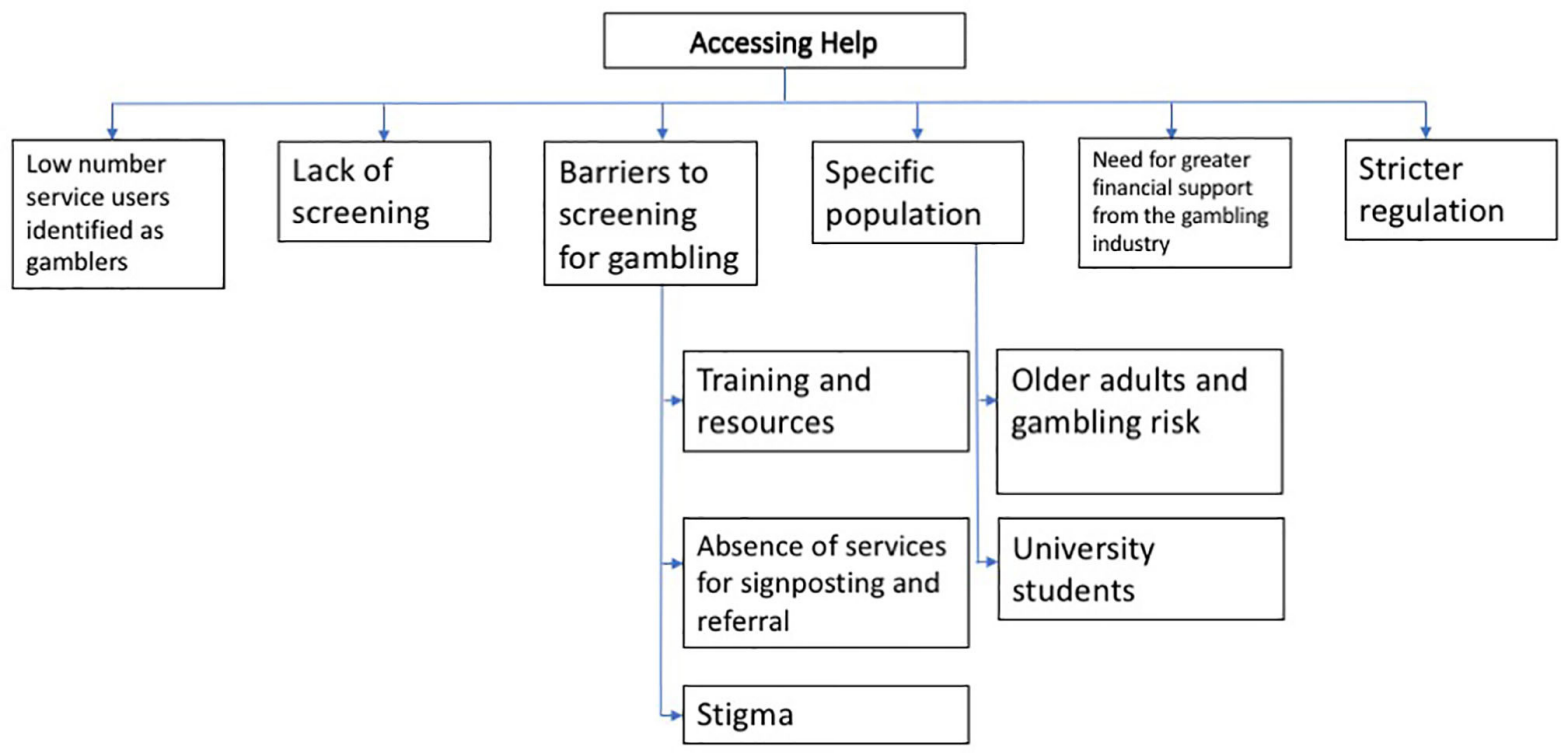
Main themes from Service Provider interview data.

Results of the content analysis of the service provider data are set out in Figure 2 below. Verbatim responses are presented with the code “SP” and relevant findings are discussed in relation to the research questions.

#### Case Study In-depth Interview Data

**Figure 3:**
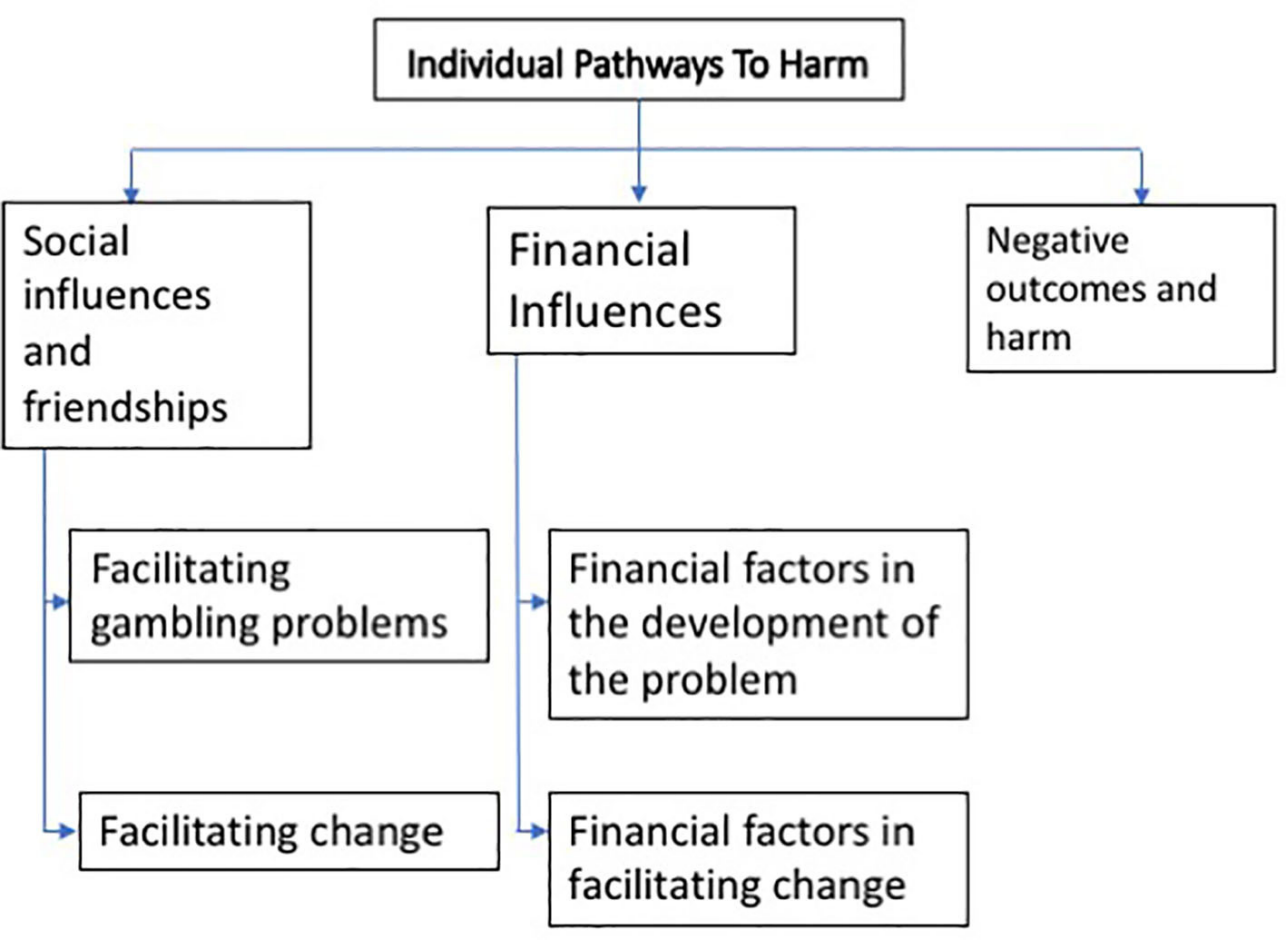
Emergent themes from Case Study interview data.

Emerging themes from the narratives of the case study interviews are set out in Figure 3 below. The code “CS” was assigned to verbatim responses from the case study interview data, which are presented in relation to relevant research questions in the results.

### Gambling Outlets Density and Availability Data

Data were geocoded in “Doogal,” a GIS that allows postcodes and density to be mapped visually. Socio-economic characteristics of the areas in which the LGOs were located were established by matching the postcode of the LGO location to its respective LSOA.

## Results

The results are set out under the specific questions posed:

### Question 1: What Are the Current Patterns, Trends, and Risks in Gambling Behavior in a General Population?

#### Patterns of Gambling and Risk Behavior in a General Population

The most frequent gambling activities in this sample were the National Lottery; slot machines; and online betting on sports and other events. Least common is gambling in traditional physical settings (bingo halls and casinos). The vast majority of respondents were able to name a wide range of gambling brands (maximum 16, mean 4). Survey responses suggest discrepancies in how people define what constitutes a “gambler” or identify their personal relationship to gambling: 26% did not consider themselves to be gamblers, but 99% reported engaging in gambling activities of some sort.

Twenty seven percentage of respondents reported some gambling risk indicators as measured by the PGSI: 15% low risk, 7% medium risk and 5% high risk. Over 50% of respondents said that they gamble alone. 40% were classified on the FAST Alcohol Screening Test as drinking at hazardous levels, with most drinking taking place at home.

Gender differences in gambling frequency were assessed using a one-way multivariate Analysis of Variance which indicated that men gambled more frequently than women [F_(8, 195)_ = 4.73, *P* < 0.001; Wilks λ = 0.84]. Univariate ANOVA revealed that with the exception of the national lottery [*F*_(1, 202)_ = 0.526, *p* = 0.469, η^2^ = 0.003] and playing in bingo halls [*F*_(1, 202)_ = 0.217, *p* = 0.642, η^2^ = 0.001], women were found to gamble significantly less frequently than men in all gambling behaviors: Slot machines [*F*_(1, 202)_ = 4.532, *p* = 0.034, η^2^ = 0.022]; Virtual gaming bookies [*F*_(1, 202)_ = 17.594, *p* < 0.001, η^2^ = .080], Table games/casino [*F*(1,202)= 3.671, *p* = .057, η^2^ = .028]; Online games [*F*_(1, 202)_ = 7.803, *p* = 0.006, η^2^ = 0.037]; Online betting/sport events [*F*_(1, 202)_ = 22.577, *p* < 0.001, η^2^ = 0.101]; Book maker/ betting event [*F*_(1, 202)_ = 4.077, *p* = 0.045, η^2^ = 0.020]. Univariate ANOVA also revealed that women were also less likely to gamble whilst intoxicated [*F*_(1, 172)_ = 20.122, *p* < 0.001, η^2^ = 0.105] and had lower overall hazardous drinking levels [*F*_(1, 176)_ = 4.797, *p* = 0.030, η^2^ = 0.027]. See Table 1.

**Table 1:** Mean (SD) gambling frequency indicators and hazardous drinking score by gender.

**Gender**	**Male**	**Female**
National Lottery^*^	0.98 (1.16)	1.09 (1.08)
Bingo Halls^*^	0.19 (0.51)	0.23 (0.63)
Slot Machines^*^	0.55 (0.93)	0.31 (0.68)
Virtual gaming bookies^*^	0.47 (0.98)	0.07 (0.34)
Table games/casino^*^	0.30 (0.69)	0.13 (0.56)
Online games^*^	0.76 (1.29)	0.33 (0.90)
Online betting/sport events^*^	0.93 (1.34)	0.26 (0.60)
Book maker/ betting event^*^	0.53 (1.06)	0.30 (0.57)
Gamble whilst intoxicated^**^	0.34 (0.66)	0.04 (0.19)
Fast Total	2.94 (2.74)	2.10 (2.37)

* Frequency scale: 0 “Never” to 4 “Daily or almost daily”;

** *Frequency scale: 0 “Never” to 3 “Always”*.

#### The Relationships Between Key Gambling and Other Risk Behaviors

Correlational analyses were conducted to assess the relationship between key risk variables and gambling harm (see Table 2 below). Risk of problem gambling and impaired control was highly correlated with frequency of gambling behavior, gambling brands recognition, hazardous drinking, intoxicated gambling, and motivation to gamble (especially gambling for excitement and gambling as a coping strategy). Individuals who reported drinking at a hazardous and harmful level, reported gambling more frequently, having less control of their gambling, and had high motivation to gamble.

**Table 2:** Relationships between key gambling and risk behaviors.

	**Age**	**Fast t**	**m Enhance**	**m Cope**	**m Social**	**PGSI**	**Gambling freq**	**Brand**	**Intox gamb**
Age	1								
FAST T	−0.200^**^								
M enhancement	−0.204^**^	0.312^**^							
M cope	−0.139	0.269^**^	0.670^**^						
M social	−0.170^*^	0.299^**^	0.543^**^	0.363^**^					
PGSI	−0.166^*^	0.303^**^	0.647^**^	0.789^**^	0.367^**^				
Gambling freq	−0.164^*^	0.318^**^	0.757^**^	0.704^**^	0.537^**^	0.683^**^			
Brand	−0.272^**^	0.297^**^	0.492^**^	0.330^**^	0.243^**^	0.356^**^	0.484^**^		
Intox gamb	−0.295^**^	0.298^**^	0.483^**^	0.442^**^	0.388^**^	0.407^**^	0.494^**^	0.268^**^	1

Intox Gamb, Intoxicated gambling score; PGSI, Risk of impaired control of gambling score; Gambling Freq, frequency of gambling score; M Cope, coping mechanism as a motivation for gambling; M Social, Social factors as a motivation for gambling; M Enhance, enhancement/excitement as a motivation for gambling; Brand, number of gambling brands recall; Fast T, hazardous drinking score. (Correlations were significant at

* p < 0.05;

** *p < 0.01 levels)*.

Younger people reported higher levels of alcohol consumption, were more likely to gamble when intoxicated and had a higher risk of problem gambling. Age impacted less on people's motives for gambling, but for younger participants the strongest motive was enhancement or excitement, whereas gambling as a coping strategy was found to be more consistent across age groups.

### Question 2: What Are the Salient Social, Cultural, and Environmental Factors in the Development of Gambling Problems?

In order to explore specific drivers or predictors of harm, a series of multiple regression analyses were conducted using the Enter method. The values of the residuals in each model were not normally distributed, however. While only extreme deviations from normality are likely to have an impact, an element of caution should be applied to interpreting these results in isolation. All other assumptions were met and no collinearity issues were identified. Results from the qualitative data also provided further insight.

#### Risk Behaviors as Predictors of Higher Levels of Gambling

Drinking patterns, motives for gambling (enhancement/excitement, social and coping reasons), intoxicated gambling frequency and age were included in the regression model as possible predictors of frequency of gambling. A significant model was found [*F*_(6, 167)_ = 56.415, *p* < 0.001, Adj *R*^2^ = 0.66] with motives for gambling emerging as significant predictors of gambling frequency (Enhancement/excitement: β = 0.401, *t* = 5.87, *p* < 0.001; Social: β = 0.158, *t* = 2.88, *p* < 0.01; Coping: β = 0.328, *t* = 5.36, *p* < 0.001).

#### Risk Behaviors Most Likely to Predict Gambling Harm

In a similar regression, drinking patterns, motives for gambling, intoxicated gambling, age and overall gambling frequency were included as possible predictors for risk of impaired control of gambling. A significant model was found [*F*_(6, 166)_ = 47.153, *p* < 0.001, Adj *R*^2^ = 0.65] with coping motives for gambling (β = 0.568, *t* = 8.50, *p* < 0.001) and Frequency of Gambling (β = 0.191, *t* = 2.45, *p* < 0.05) emerging as significant predictors. This finding suggests that using gambling as a means of coping with negative issues in one's life is a highly risky strategy, and likely to lead to problematic gambling behavior.

#### Gambling Behaviors Which Predict Gambling Harm

Frequency of engagement with all methods of gambling measured were included as possible predictors of risk of impaired control of gambling. A significant model was found [*F*_(8, 169)_ = 40.50, *p* < 0.001, Adj *R*^2^ = 0.64] with Fixed Odds Betting Terminals (FOBTs) in Licensed Gambling Outlets (β = 0.438, *t* = 7.82, *p* < 0.001), and online betting on sports events (β = 0.473, *t* = 7.99, *p* < 0.001) emerging as significant predictors. Individuals who engage in these types of gambling are much more likely to experience risk of problems in their gambling behavior.

#### Qualitative Survey Findings on Patterns and Predictors of Harm

Qualitative data was analyzed to explore patterns and predictors of gambling harm. The first three overarching themes that emerged from the qualitative survey data; Gambling Harm; Drivers of Gambling; and New Technologies (and the subthemes; Figure 1), appear to corroborate the quantitative findings in terms of patterns and predictors of harm.

Findings further suggested that FOBT machines were perceived as harmful:

**“***The fixed-odds terminals became my addiction for a time - and I would bet to the maximum £100 per spin quite often”* [QS]

“*When I have gambled and won, I tend to pump all my money back into the machine until I lose*” [QS]

“*FOBT gambling drove me to attempt suicide a few years ago.”* [QS]

New technologies in the form of online and phone app gambling sites are also confirmed as major contributors to harm through ease of access, the loss of social norms and pressures, and the buffer of spending money via credit cards.

“*There are a ridiculous amount of online outlets for gambling with table games/slots… the sheer number of them operating obviously shows a massive market with high profit”*. [QS]

“*Gambling in the UK is quite a normal thing to do…… with online apps meaning that you can easily gamble on the go”*. [QS]

“*people are getting caught up in the wild west of online casino vendors where the reality of parting with physical cash is removed and people are spending ‘imaginary money”’* [QS]

The qualitative data also support the finding that coping is a common motivation for gambling and is a harmful strategy and suggest that the industry targets those who may be more vulnerable to engaging in gambling activity.

**“***Online betting apps have been taking advantage of Facebooks targeted advertising. Like the betting shops they are targeting low earners and vulnerable people”*. [QS]

“*I think people ………… mainly gamble to try and get out of poverty. I think it's a no win situation and you end up losing more than you win. But when you're desperate I can see why it's tempting”*. [QS]

#### Patterns and Predictors of Harm: Density Mapping

Further, the density mapping data appear to confirm the notion (which has emerged from the qualitative data) of industry targeting of vulnerable demographic groups. Three out of five sites under investigation had clusters of LGOs in their town centers that could be considered “high density,” with six or more located within a 400 m radius. By mapping nationally available indices of socio-economic and demographic status onto the locations of the LGO clusters, we were able to establish these to be disproportionally distributed in areas of social-economic deprivation. In two of the sites LGOs were located across the most socio-economically deprived LSOAs of the towns. As such, these findings demonstrate that geographical location may play a role in the risk of gambling related harm (see Table 3).

**Table 3:** Descriptive results for density mapping of LGOs.

**Location (Licensed Authority)**	**Type of LGO**				**LGO rate per 10,000 in population**
	**Bookmakers**	**AGCs**	**Bingo Halls**	**Total**	
RCT	39	6	3	48	2.0
Denbighshire	18	6	7	31	3.2
Newport	22	4	1	27	1.8
VoG	10	12	–	22	1.7
Wrexham	16	3	1	20	1.4

*RCT, Rhodda Cynon Taff, VoG, Vale of Glanmorgan*.

### Question 3: Do the Personal Accounts of Individuals (Gamblers and Their Families/Friends at the Problem Gambling End of the Scale) Have Salience With Wider Patterns and Trends in Terms of Risk Predictors?

It is apparent that the experiences of individuals further along the harm continuum reflect, and thus further corroborate, the trends and patterns identified in the quantitative data as “upstream” predictors. Such predictors include social, cultural, and environmental factors.

#### Social Factors

A key theme in the case study accounts was the importance of “social influences and friendships” in both facilitating gambling behavior and the development of future problems. These narratives appear to reflect the significant predictors of harm that emerged from the multiple regression analyses in the centrality of social and excitement motivations for gambling, specifically in younger people. Four of the five individuals interviewed recalled their early gambling experiences as involving social occasions with friends, particularly their desire to be part of the group, but also the excitement of seeing their friends win considerable amounts of money.

“*First time I went with my friend, in to the bookies, I was in the army. He had been before but it was my first time” [CS1]*

Participant five was introduced to gambling whilst playing snooker with friends at the age of 16, and very quickly was spending more money than them on the fruit machines in the snooker hall, “*twenty pounds a time, that was a lot of money back then” [CS5]*

Participant two reflected that watching his friends win large amounts of money was one of the things he initially found appealing about gambling. He sees that his gambling behavior became financially motivated overtime, but the initial motivation was to fit in with his friends.

“*. it was a combination of the financial and the fitting in. I don't want to single out one.”[CS2]*

Participant three discussed in detail how his social circle and friendship groups influenced his gambling. He believes that he first began gambling because it was something his friends did and he would rarely gamble alone. Over time, he found himself moving from relatively low risk gambling (football accumulators) to more high stakes betting (roulette), and he attributes this increase to his friends who introduced him to the games.

“*So when I was at uni. my mates they would go to do football (accumulators) but they would go on to these roulette machines. I didn't understand the machines but when I was with them I would see them make quite a lot of money” [CS3]*

As such, the progression from low risk betting to higher risk betting demonstrates a movement along the continuum of gambling related harm/risk behaviors, which originated as gambling in a social environment.

#### Cultural Factors

Culturally, there is a hidden element to much gambling behavior, which may be an important factor in the development of harm. The majority of respondents to the survey reported gambling alone, a pattern that is facilitated by developments in technology. Additionally, changes in legislation appear to have contributed to changes in behavior patterns, as is the case with alcohol. In this sample, most alcohol was reported as consumed at home, and the quantitative data suggest that there is a strong relationship between drinking and impaired control of gambling. Qualitative responses support these associations:

Bingo in a community hall is a social activity, whereas bingo online is an addictive activity that feeds gamblers - it's dangerous [QS]

Online bingo is just as serious a problem as the casino websites, but doesn't seem to get anywhere near the moral backlash [QS]

“*I've always gambled alone. It was always virtual though – I've never gone over to bookies or even events.” [CS4]*

“*I know how closely linked drinking and gambling is as have had a close family member suffer. It has a huge effect on the near family and alienates many people. It can be hidden until it gets out of hand” [QS]*

The hidden nature of gambling is also evident in the reported reluctance to admit to gambling problems, possibly because of denial, associated stigma or a lack of self-awareness, as documented in responses of gamblers experiencing problems, and their family members:

“*my best mate [of 40 years] said ‘you've got a problem with those machines’. I just sort of dismissed it at the time, but I thought about it later and I thought maybe he's right”. [CS5]*

“*I covered it up for a long time [from his wife], but one day it came to a head. I was fed up of lying to her and covering my traces.” [CS5]*“*It kills families. And it's one that's hidden, and usually not helped” [QS]*

This pattern was also observed by service providers (within the theme of “barriers to screening for gambling,” see Figure 2):

“*It could be useful to ask these [about gambling behavior] questions as many people may not realize that they have any issues with gambling or even that they are gambling.” [SP]*

“*someone with a gambling addiction can be very closed off about the subject and may not even be able to admit they have a problem to themselves.” [SP]*

“*it is extremely rare that a client will say their debts are as a result of gambling.” [SP]*

“*Clients rarely bring up gambling problems for various reasons ranging from embarrassment to not realizing there are any problems with their behavior.” [SP]*

#### Environmental Factors

Rapid changes in gambling environments also point to new “at risk” groups being targeted. A strong theme within the survey qualitative data relates to the gambling industry's advertising strategies:

“*the way betting companies market it as a fun exciting activity is dangerous” [QS}*

“*a lot of advertising for betting online [is] being portrayed as an attractive past time and fun thing to participate in, rather than gambling” [QS]*

Further, there is a sense that being able to gamble online removes the **“*stigma effect”*** of being seen coming out of the Licensed Gambling Outlet, as well as the associated negative stereotypes:

“*A lonely old man in the betting shop – on line betting using apps is portrayed as ‘cool”’ [QS]*

“*when people are home alone there is no pressure on them, not like being seen coming out of bookies. Very, very dangerous” [QS]*

Service provider accounts support this, with a number reporting that they are identifying specific populations who may be at higher future risk due to targeted gambling promotion. These include older adults, whose vulnerabilities may be targeted in different ways from younger gamblers:

“*We have anecdotal evidence that older people are susceptible to gambling promotions that may help them feel engaged and important at times of vulnerability and loneliness.” (SP)*

Certain student groups are also seen as more at risk:

“*I have noticed that sports students are particularly at risk. A lot of ads target them, we've noticed it's quite prolific in sports students.” (SP)*

“*Isolated students are using [certain gaming sites] for friendship. Some of these sites have chat functionality and people use them for friendship too.” (SP)*

Mature students who may “*put the kids to bed and then go on the bingo sites for a couple of hours” (SP)*

Thus, the findings from qualitative accounts demonstrate salience between the personal accounts of individuals and the patterns observed in the upstream predictors of gambling harm.

### Question 4: How Effective Are Support Services in Identifying and Helping Individuals With Gambling Problems?

In the absence of specialist services in the geographical region under investigation, “proxy” service providers were recruited (see methodology). With the exception of the very few specialist service providers interviewed, the overwhelming majority noted that they very rarely came into contact with service users with an apparent gambling issue. However, these particular services do not formally screen for problem gambling, or indeed raise the issue informally with clients, despite the latter often presenting with financial situations that might indicate a problem. Concern was expressed that simply asking about gambling behavior could raise false expectations in service users that the organization was equipped to help them as service providers do not believe that they have the skills or resources to be able to help if a gambling problem is identified.

“*Training would be required on how to deal with people who do disclose” [SP]*

“*I do feel that questions regarding problem gambling should only be asked if there is immediate support that can be offered to the client in regards to this.” [SP]*

“*This [asking about gambling behavior] could lead to confusion for the service users as they believe that we are able to offer support around their gambling” [SP]*

The lack of routine screening and assessment in identified proxy services results in an absence of systematic data to both quantify need, and to inform service development. The reluctance to screen is to some extent predicated on a lack of services to refer on to, which illustrates the circular nature of this issue, with the resultant difficulty in both establishing a “line of harm” and a continuing dearth of treatment options. Findings suggest, however, that it is possible to break this circle through reducing the identified barriers. Service providers working in the context of students' services reported positive developments through receiving basic training in screening for gambling issues in students;

“*We are more aware and more confident. We're not specialists, we are student money advisors, but this is a stepping stone to confidence to ask about gambling behavior” [SP]*

The one service provider with a specialist gambling intervention service is clear on the need after conducting their own brief scoping exercise, describing it as

“*like lifting a scab on the huge hidden problem” [SP]*

The experiences of the individual case study interviewees appear to confirm the lack of support services. A common experience was difficulty in finding help once they had acknowledged that they had a problem. Searching online (for help services) was a common starting point, and for some, helpline advice was the extent of the help they had received. Two respondents were receiving counseling with trained therapists, and both felt that this was contributing to their recovery and relapse control.

“*I found Gamblers Anonymous, I looked online” [CS5]*

“*I didn't find anyone that could help me…. I researched a bit and there were some support groups but they were like, multiple people who have gambled. I eventually found this place [gambling support counseling service], and it took me a year and a half. It's not obvious if you need help, it's not easy access”. [CS2]*

## Discussion

### Objectives of the Current Study

The objective of the current study was to contribute to the development of a Public Health Framework for addressing the issue of gambling harm, through investigating steps 1 and 2 of the WHO four-step model: (1) understanding the nature and extent of the problem, and (2) identifying the causes. A multi-method approach (including survey, structured interview, and case study approaches) was utilized: to gather data relating to individuals presenting in treatment programmes and other services; to explore the broader, “upstream” gambling patterns and trends; and to map the geographical density of and access to gambling outlets. The qualitative and quantitative findings overlap and corroborate, strengthening the emerging narrative on the conception of a continuum of harms associated with gambling problems, and the relevance of this to a population level approach to harm reduction.

### Nature and Extent of the Problem

In relation to patterns and trends upstream, the current study finds 27% of respondents demonstrating some gambling risk indicators as measured by the PGSI. It should be noted that the survey participants were individuals with some interest in gambling, and therefore not necessarily representative of the population as a whole. Thomas et al. (8) reported a slightly higher 40% of respondents with some gambling risk indicators in Australia, and whilst the authors acknowledge the possibility of over-representation of gamblers in their sample, they also point out that the findings were similar to those of community-based surveys. That nearly a third of the sample is evidencing some risk of harm, in a general rather than clinical sample, suggests further support for a continuum of gambling related harm, as opposed to the established notion of a dichotomy of social vs. risky gambling behavior with the latter affecting a small minority of individuals [see for example, Browne et al. (28)]. The most frequently reported types of gambling behavior (excluding the National Lottery) are FOBT gaming machines in LBOs and internet/phone app-based sports betting. The levels of engagement with the latter are higher than reported in the last Welsh population survey (29), which may reflect the increasing ease of access and availability of new gambling products, as well as improved internet connectivity. In this study, FOBTs and gambling online on sports' events were the significant predictors of harmful gambling behavior. These specific types of gambling are increasingly technology-driven, solitary and/or away from the public arena, factors that combine to suggest a clear potential for a “concertina effect” in terms of the speed of harm development (30).

### Social, Cultural, and Environmental Contexts/Causes

An understanding of the influence of social, cultural and environmental contexts is central to the development of a public health approach to gambling harms, evidenced in the taxonomy of gambling harms developed by Langham et al. (10). An in-depth review of the emerging international literature is beyond the scope of this paper, but these are overlapping constructs, as the triangulated data from the current study demonstrate, with strong relationships between the majority of the risk behaviors measured. The findings suggest that alcohol consumption and drinking patterns play a part in impaired control of gambling, with younger gamblers more likely to consume more alcohol and also to gamble when intoxicated. Motivational drivers are significant factors in both gambling frequency and harm. Excitement and social motivations are stronger in younger gamblers and are highly predictive of gambling frequency, which is itself a predictor of risk of harm. Castren et al. (31) report similar findings in relation to men. Further, the importance of social factors in both the initiation and maintenance of gambling emerges as a strong theme in the case studies of individuals who have experienced gambling problems. The majority cite the early influence of friends in both gambling and winning money as a common attribution for their subsequent development of gambling related harm; the former is supported by a number of studies in relation to gambling [for example, (32)] and other health risk behaviors such as alcohol consumption [for example, (33)].

The motivation of gambling as a coping strategy does not appear to be age related and seems to be strongly predictive of problems and harm. This aligns with the qualitative data theme of cynicism about the gambling industry, particularly participants' perceptions that industry advertising targets poorer populations who may be more susceptible to the false hope of escaping desperate socioeconomic situations. Recent studies on the impact of marketing indicate that riskier gamblers are more attuned to and thus impacted by advertising [for example (15)], although Browne et al. (16) found this effect across all types of gamblers. The density mapping data would also appear to confirm the notion of high environmental risk, with clusters of LGOs and FOBT machines disproportionately high in these areas.

The broader cultural context of gambling harm appears to parallel that of alcohol in previous decades, particularly in relation to issues of definitions of problems, denial and stigma. The survey data identified a discrepancy between how people define what constitutes a “gambler” and their personal relationship to gambling, with 26% not considering themselves to be gamblers, despite nearly all participants reporting engaging in gambling activities of some sort. This also emerged as a subtheme in the case studies in terms of denial of problems and a reluctance to seek help, which was further corroborated by the interviews with service providers, with many highlighting ignorance, reluctance and embarrassment in people to admit to gambling related problems. Stigma or negative stereotypes may be drivers of a lack of openness in relation to problem development, but there may also be lack of insight into one's own gambling activity (i.e., perception of self-vs. others), which is often evident in individuals who consume alcohol at hazardous levels (33). These may be important considerations in the development of effective harm reduction messages. Similarly, issues around stigma appear to be important barriers to identification of need by “proxy” services, where service providers report a reluctant to screen for gambling problems. This position, and the reasons given by the service providers (e.g., lack of time, skills and resources to deal with identified problems), resonate with early exploration of the potential role of Primary Care health professionals in identifying alcohol problems (34). Reducing such barriers is essential for the development of early interventions for problem gambling. A public health approach that reframes the issue to a continuum of harm, as opposed to pathologizing a minority of “problem” gamblers, could change the acceptability of acknowledging the need for help by both gamblers and service providers.

### Strengths and Limitations

A limitation of the current study may be that the study was conducted solely in Wales, and thus questions of generalisability may be raised (due to specific contextual factors such as SES and local culture.). However, this could also be seen as a strength, in that this is the first study to explore gambling harms in the Welsh context. These findings support emerging evidence from other countries, including Australia, Spain, China and Japan (8, 18, 19, 35) and as such contribute to the wider international literature and debate on gambling related harm. Further, whilst it is clear that there are universal issues in identified gambling harm, social, cultural and environmental contexts will differ across geographical regions. Gambling is both a local and global public health concern. Building a rigorous international evidence base will strengthen the arguments for national and local legislative and policy change.

## Conclusion

In conclusion, as initial steps in the development of a public health framework for gambling harm, this study has: (1) explored the nature and extent of the problem, and (2) investigated identifying causes. In line with findings from Australia, Japan and other countries, the results support the notion of a continuum of harm and identify a complex and interrelated set of drivers or predictors of future problems. There is clearly a need for action at a policy/legislative level (as evidenced in the disproportionate density of LBPs in areas of low SES and identified targeting of disadvantaged communities). It is also clear that broadening the focus of intervention from individual pathology to a population level public health approach is necessary to develop effective strategies for harm reduction requirements at all points. Further, to some extent, the findings of this study support Babor et al.'s (11) assertion that an understanding of the heterogeneity of sub-populations is important in considering a public health approach to gambling, in that there appear to be differences in age related gambling behaviors and related risk. Future research should further investigate demographic differences in risk, harm and engagement in new gambling products in this rapidly developing industry.

## Data Availability Statement

The raw data supporting the conclusions of this article will be made available by the authors, without undue reservation, to any qualified researcher.

## Ethics Statement

The studies involving human participants were reviewed and approved by Ethics committee for the Faculty of Life Sciences and Education, University of South Wales. The patients/participants provided their written informed consent to participate in this study.

## Author Contributions

BJ and GR-D led all aspects of this work from design, data collection, and analysis to overseeing manuscript preparation. KH was involved in the design and interpretation of this work. ND, MB, and TM contributed to the design, data collection, and analysis of this work. ST and AC contributed to the analysis and interpretation of the work and to manuscript preparation. All authors contributed to the article and approved the submitted version.

## Conflict of Interest

The authors declare that the research was conducted in the absence of any commercial or financial relationships that could be construed as a potential conflict of interest.

## Footnotes

^1^FOBTs are categorized as either B2 or B3 machines under the 2005 Gambling Act.

^2^The licensing authorities for each site are; Rhondda Cynon Taff County Borough Council, Wrexham County Borough Council, Newport City Council, Vale of Glamorgan Council and Denbighshire County Council.
